# Text Embedded Swin-UMamba for DeepLesion Segmentation

**Published:** 2025-08-08

**Authors:** Ruida Cheng, Tejas Sudharshan Mathai, Pritam Mukherjee, Benjamin Hou, Qingqing Zhu, Zhiyong Lu, Matthew McAuliffe, Ronald M. Summers

**Affiliations:** a Scientific Application Services, Center of Information Technology, NIH; b Division of Intramural Research (DIR), National Library of Medicine (NLM), NIH; c Imaging Biomarkers and Computer-Aided Diagnosis Laboratory, Radiology and Imaging Sciences, Clinical Center, NIH

**Keywords:** Universal Lesion Segmentation, DeepLesion

## Abstract

Segmentation of lesions on CT enables automatic measurement for clinical assessment of chronic diseases (e.g., lymphoma). Integrating large language models (LLMs) into the lesion segmentation workflow offers the potential to combine imaging features with descriptions of lesion characteristics from the radiology reports. In this study, we investigate the feasibility of integrating text into the Swin-UMamba architecture for the task of lesion segmentation. The publicly available ULS23 DeepLesion dataset was used along with short-form descriptions of the findings from the reports. On the test dataset, a high Dice Score of 82 ± 18% and low Hausdorff distance of 6.58 ± 10.64 (pixels) was obtained for lesion segmentation. The proposed Text-Swin-UMamba model outperformed prior approaches: 37% improvement over the LLM-driven LanGuideMedSeg model (*p* < 0.001), and surpassed the purely image-based xLSTM-UNet and nnUNet models by 1.74% and 0.22%, respectively. The dataset and code can be accessed at https://github.com/ruida/LLM-Swin-UMamba

## 1. INTRODUCTION

Lesion assessment on CT is essential for early diagnosis, treatment planning, and longitudinal monitoring of chronic diseases, such as lung and liver cancer. ^1,2^ In current clinical practice, lesion size and characteristics (e.g., enhancement, attenuation attributes, irregularity) play a predominant role in the determination of malignancy of suspicious findings^3,4,5^. For example, the management of liver lesions follows the sizing guidelines of the American College of Gastroenterology^1^, while lung nodules are to be managed using an entirely different set of sizing guidelines from the Fleischner Society^2^. Accordingly, radiologists measure the long- and short axis diameters (LAD and SAD) of lesions, which serves as surrogate marker for malignancy. Moreover, it is cumbersome for radiologists to manually size the dimensions of several lesions during the busy clinical day, especially when faced with metastasis. Other confounding elements are the acquisition of CT studies on myriad scanners from different manufacturers and the diverse appearances and shape of lesions in these studies. Despite these variations, radiologists must dictate the size and characteristics of suspicious lesions into a report.

While several prior works for lesion segmentation have been proposed, few have investigated the utility of lesion descriptions present in the reports. In natural image domain, many prior works have incorporated Large Language Models (LLMs) for image segmentation or object detection tasks.^6,7,8^ However, porting their work to the medical image domain poses a significant challenge due to suboptimal segmentations as organs or tumors often have lower contrast, ambiguous boundaries, and complex anatomical relationships. Our approach does not rely on existing mainstream foundation models, such as ChatGPT^9^, because they are not open-sourced and cannot directly be trained or fine-tuned for task-specific medical image segmentation. An early study, Liu et al.^10^ explored a Contrastive Language Image Pre-training(CLIP)-driven universal model to tackle CLIP embedding for organ labels. Li et al.^11^ reported a text-augmented Transformer model for segmentation, L-ViT, while Zhong et al.^12^ used GuideDecoder and text prompts to enhance the segmentation results. All three architectures, in general, encoded text into the segmentation backbone, either guiding or improving the prediction of segmentation masks. Importantly, all three approaches missed the inclusion of a text decoder component due to the complexity of the relational memory-based architecture.^13^ Previous works by Yan et al.^14,15^ combined lesion features and short-form report text descriptions to learn relationships among related lesions, attributes, and surrounding anatomy.

In this study, we investigate the feasibility of adapting text-based embedding directly for lesion segmentation. We present the Text-Swin-UMamba model that implements multiple language-embedding mechanisms (Text Tower^19^ and LLaVA-style^23^ encoder) to embed short-form report text descriptions into the decoder of Swin-UMamba^14^ for lesion segmentation. The model incorporates text features from the report text at multiple decoder stages, improving contextual understanding during lesion segmentation. Our method was compared against prior approaches (LanGuideMedSeg^13^, xLSTM-UNet^20^, and nnUNet^21^) and demonstrated improved lesion segmentation performance through fusion of text and imaging features.

## 2. METHODS

**Figure 1.**
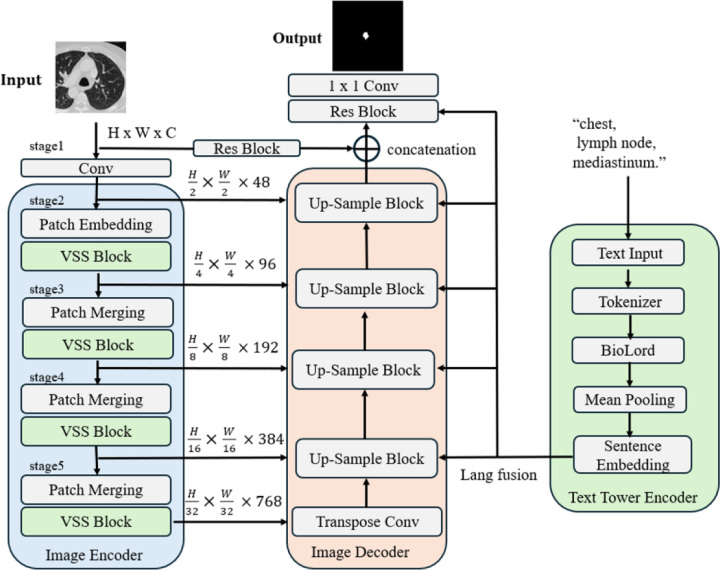
The design of Text-Swin-UMamba where the Text Tower encodes short-form descriptions of clinical findings on 2D slices in the DeepLesion dataset and embeds them into the Swin-UMamba segmentation backbone.

The overview of the proposed Text-Swin-UMamba architecture is shown in Figure 1. The model is composed of three major building blocks: the Swin-UMamba image encoder, the Swin-UMamba image decoder, and the text tower encoder. We follow the Swin-UMamba implementation, a derivative of the fully automated and self-configuring nnU-Net framework, as the segmentation backbone of the proposed model. For the text embedding, we utilize the Text Tower^19^ encoder to convert short-form text from the radiology reports into textual feature representations. The Text Tower building block aims to take a text report as input, tokenize it, pass it through a pre-trained “BioLord” model, and then produce a pooled (summarized) embedding for each text input. The Tokenizer layer serves as a custom text preprocessing utility. The BioLord pretrained model can produce meaningful representations for clinical sentences. The mean pooling layer obtains a fixed-size embedding from the variable-length token embedding produced by the BioLord tokenizer. Mean pooling layer calculates the average of all the contextualized token embeddings. The final output of the Text Tower is a fixed-size vector (e.g., 768 dimensions) that represents the semantic meaning of the entire input text report.

The language features are then integrated into the Swin-UMamba decoding path at multiple scales, particularly at five stages of the decoder, corresponding to feature maps (Figure. 1). The language feature is passed through a dedicated linear projection layer (Lang Fusion) to match the channel dimensions of the respective image features. The multimodal fusion mechanism between the Text Tower encoder and the Swin-UMamba decoder can guide the Swin-UMamba with text-based semantic information. A few other text encoding and fusion mechanisms are also implemented, such as LLaVA Text Tower, Bert-based encoding and fusion, and tail encoding (Please refer to Github repository for implementation detail).

## 3. EXPERIMENTS

### Dataset.

The ULS23 DeepLesion dataset^17^ was utilized as validated labels were available in this dataset. The dataset tailored from the original DeepLesion dataset, which contained 4427 unique patients across 10,594 studies, and 32,120 CT image slices (age: 57 ±18 years, 56% males, 44% females). The structured text description corresponding to a finding on a specific 2D slice in the original DeepLesion dataset (and annotated in the ULS23 dataset) was extracted. Each sentence provides detailed information, including its location (e.g., left lung, retroperitoneum), lesion type (e.g., nodule, mass, lymph node), and relevant attributes (e.g., hypoattenuation, enhancement, irregularity). The rationale for the choice of this dataset was due to the limited availability of datasets with annotated lesion and corresponding report text. For example, Qiu et al.^16^ did not release their lesion benchmark dataset, SegLesion. Each 2D CT image slice is 512×512 pixels in size. A significant number of lesion masks appear in a small or tiny region of the image slice, posing substantial challenges for direct lesion segmentation. The 2D CT image, its corresponding label from the ULS23 DeepLesion dataset, and the sentence description constituted our dataset, which was divided into training (n = 15040), validation (n = 3760), and testing (n = 1807) data subsets, respectively. The dataset was divided at the patient level with no overlap across the splits.

### Comparisons.

The proposed LLM-Swin-UMamba method was compared against prior approaches including LanGuideMedSeg ^12^, xLSTM-UNet ^20^, and 2D nnU-Net ^21^.

### Implementation Details.

Five-fold cross-validation is conducted in training and validation stage. All models are trained on a single NVIDIA V100x GPU with 32 GB of memory. The loss function integrates the Dice coefficient loss with the cross-entropy loss. AdamW optimizer with initial learning rate of 5e-3, scheduled via cosine anneal. The training epochs are set for 1500. The complete training phase for each model takes approximately 3 to 5 days to complete.

## 4. RESULTS

**Figure 2.**
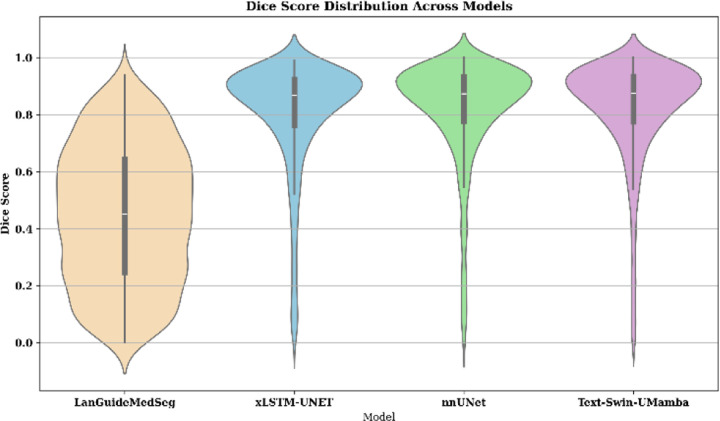
Distribution of Dice scores for each model on the ULS23 test dataset

**Figure 3.**
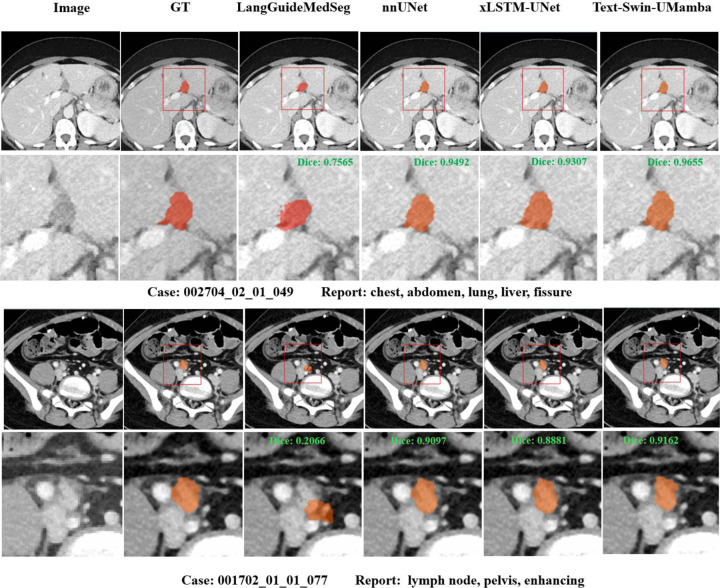
Qualitative results of segmentation of a liver lesion and a lymph node by the proposed Text-Swin-UMamba model compared against the other approaches.

Table 1 describes the results of the various models on the test dataset, while Figure 2 shows the distribution of Dice scores. Figure 3 details qualitative segmentation results overlaid on the CT. The proposed Text-Swin-UMamba model improved the Dice score by 37% (0.82 vs. 0.45, *p* < 0.001) over the LanGuideMedSeg model. Against other models without text embedding, it outperformed the xLSTM-UNet model by 1.74% (0.82 vs. 0.8, *p* < 0.001) and achieved a slightly higher mean Dice score of 0.22% (*p* > 0.05) compared to the 2D nnU-Net model. Notably, the model obtained a lower HD error than the 2D nnU-Net (6.58 vs. 6.86 pixels).

## 5. DISCUSSION AND CONCLUSION

The proposed Text-Swin-UMamba model incorporated the Text Tower embedding mechanism into the Swin-UMamba multi-scale decoder to facilitate lesion segmentation. Our model achieved the best segmentation results with a mean Dice score of 81.76% and HD error of 6.58 pixels. It surpassed a similar model with text embedding, LanGuideMedSeg, by 37% (*p* < 0.001), while also posting moderate and consistent gains over purely image-based segmentation models, such as xLSTM-UNet (1.74%, *p* < 0.001) and nnUNet (0.22%, *p* > 0.05).

Our results indicate that incorporating short-form text descriptions of findings from the radiology reports can yield a noticeable advantage for lesion segmentation. The clinical context provided by both the imaging and text features guided the network to yield good segmentation results. However, our work has limitations. First, the experimental design utilizes the ULS23 DeepLesion dataset, where lesions are centered on 256×256 images to simplify the segmentation task relative to the original 512×512 images from the original DeepLesion dataset. Such specific design for the dataset constrains the generalizability of our proposed model to more realistic scenarios, where lesions are small compared to surrounding anatomy. Second, other architectural variations, such as a decoder for long-form text generation or relational memory modules^13^, have not been investigated due to their implementation complexity. Third, our approach is currently designed only for 2D images. While it can be easily extended to 3D volumes, this has not been attempted in this pilot work. In summary, Text-Swin-UMamba presents a simple approach to integrate language-guided reasoning for lesion segmentation. The text embedding mechanism can be applied to any nnU-Net model (and its derivatives). We open-source our dataset and code for further research. Future work is targeted towards scaling the approach to full-size CT volumes and enabling long-form descriptions of findings to pre-fill into the reports ^22^, which may enhance the explainability of lesion segmentation.

## Figures and Tables

**Table 1.** Testing phase comparison of segmentation models performance with the DeepLesion dataset.

	Mean Dice	Jaccard	Hausdorff95 (px)	Sensitivity	Specificity	Text Embed
LanGuideMedSeg ^12^	0.45 ± 0.24	0.32 ± 0.21	14.86 ± 15.29	0.47 ± 0.26	0.992 ± 0.023	✓
xLSTM-UNET^20^	0.80 ± 0.20	0.70 ± 0.22	7.52 ± 12.91	0.84 ± 0.19	0.997 ± 0.006	*×*
nnUNet^21^	0.82 ± 0.18	0.72 ± 0.21	6.86 ± 11.56	0.84 ± 0.19	0.997 ± 0.008	*×*
Text-Swin-UMamba	0.82 ± 0.18	0.72 ± 0.21	6.58 ± 10.64	0.83 ± 0.19	0.998 ± 0.006	✓
